# Quantitative PCCT imaging in differentiating adrenal adenomas from metastases: diagnostic performance and its clinical applications

**DOI:** 10.1007/s00261-025-04987-9

**Published:** 2025-05-21

**Authors:** Florian Haag, Shanice S. Emmrich, Alexander Hertel, Johann Rink, Abhinay Vellala, Sara Komlen, Dominik Nörenberg, Stefan O. Schoenberg, Matthias F. Froelich

**Affiliations:** Department of Radiology and Nuclear Medicine, University Medical Center Mannheim, Heidelberg University, Mannheim, Germany

**Keywords:** Photon counting computed tomography, Virtual Non contrast, Multiphasic computed tomography, Protocol optimization, Adrenal mass

## Abstract

**Objectives:**

To evaluate the potential of Photon counting CT (PCCT) -derived iodine densities and Virtual-Non-Contrast (VNC) reconstructions for the differentiation between adrenal adenomas and adrenal metastases.

**Materials and methods:**

This retrospective study enrolled 53 PCCT scans of patients with adrenal lesions (29 adenomas, 24 metastases) including early-arterial (ea) and portal-venous (pv) contrast phase. Iodine maps and VNC reconstructions were calculated from the acquired sequences. Using the measured values, several parameters such as relative enhancement (RE), normalized iodine density (NID), and iodine to VNC ratio (IVR) were calculated. In the acquired image series, iodine maps and VNC reconstructions density values were evaluated (adrenal lesion, ipsilateral adrenal tissue, contralateral adrenal tissue, portal vein, descending aorta, inferior vena cava, subcutaneous adipose tissue, in the first lumbar vertebral body, surrounding air, and in the psoas muscle) and compared.

**Results:**

In total, the measured structures showed significant variation in density values due to different contrast phases and reconstructions. VNC reconstructions of portal-venous contrast phase showed significant differences in measured density mean values between adrenal adenomas and adrenal metastases (21.64 HU vs. 28.26 HU, p: 0.027, AUC: 0.68). No significant differences between metastases and adenomas were observed in iodine concentration, RE of ea, NID of ea and pv, as IVR of ea. Significant alterations were observed in RE of pv (p: 0.023, AUC: 0.7) and IVR of pv (pv: 0.029, AUC: 0.69).

**Conclusion:**

The recent study shows that PCCT derived VNC reconstructions of portal venous contrast phase can be used for reliable differentiation of adrenal adenomas and adrenal metastases and underlines the value of PCCT in oncological imaging.

## Highlights

Photon counting CT enables Virtual-Non-Contrast reconstructions and iodine maps, which can be used for better differentiation of adrenal incidentalomas in contrast-enhanced scans of the upper abdomen.

Density values measured in Virtual-Non-Contrast significantly improve the differentiation of adenomas from metastases of the adrenal gland compared to density values measured in contrast-enhanced scans.

Additionally, several parameters, such as relative enhancement, normalized iodine density, and iodine-to-Virtual-Non-Contrast ratio, can be calculated. In particular, relative enhancement shows great potential to discriminate between adenomas and metastases.

Photon counting CT therefore adds additional capability in diagnosing adrenal glands to routine clinical workflows, providing an improved initial orientation. However, the methods still exhibit lower levels of significance and specificity compared to specialized MRI and CT protocols for imaging of the adrenal gland.

**Graphical abstract:**

## Graphical abstract

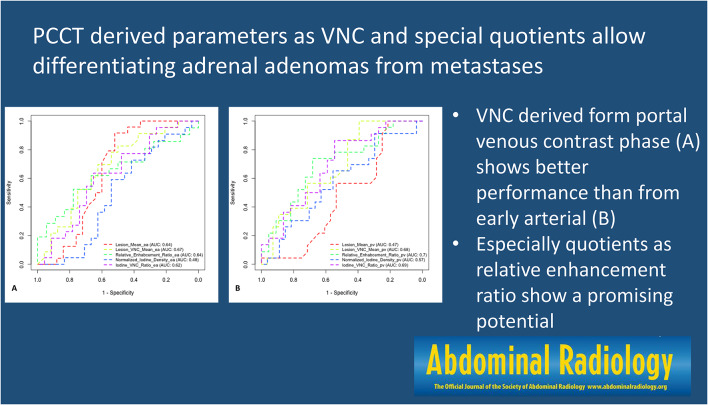

## Introduction

Adrenal incidentalomas (AIN) subsumize lesions of the adrenal gland ≥ 1 cm, which are detected incidentally during imaging of the upper abdomen [1]. The most common benign lesions are adrenal adenomas [2]. Less common benign lesions include non-malignant pheochromocytomas [3], myelolipomas [4], and cystic lesions. Examples of malignant adrenal lesions are metastases and cortical adrenal carcinoma [5, 6]. When incidentalomas are identified in CT-scans of the thorax and/ or upper abdomen, it is crucial to differentiate between potentially malignant and benign lesions.

The American College of Radiology white paper on the management of adrenal masses suggests using several imaging features for differentiating AINs. Benign features include macroscopic fat, which is typical for myelolipomas or lipid-rich adenomas, cystic lesions, haemorrhage, and calcified masses such as old hematomas or granulomatous infections [7, 8]. For precise classification of AINs, specific examination protocols for MRI and CT are recommended. MRI protocols for the adrenal gland should include chemical shift sequences for detecting intracellular fat, fat-suppressing sequences for detecting macroscopic fat, as well as T2-weighted sequences and contrast-enhanced sequences [9, 10]. CT protocols for the adrenal gland should include a non-contrast-enhanced scan, a portal-venous contrast phase (pv), and a late phase (after 15 min). These sequences are used to calculate the absolute and relative contrast agent washout of the lesions. A relative washout ≥ 40% and an absolute washout ≥ 60% are suggestive of adrenal adenomas [10–12].

If AINs are detected during clinical imaging of the upper abdomen that does not correspond to a specific adrenal gland protocol, and no clear benignity criteria are identified, a dedicated MRI or CT scan must also be performed [11]. This often requires a separate appointment and utilizes limited resources.

To address this issue, further studies have evaluated the potential of spectral imaging with dual-energy CT (DECT) to differentiate between adrenal adenomas and metastases. DECT offers Virtual-Non-Contrast reconstructions (VNC) and iodine maps, enabling better differentiation between adrenal adenomas and metastases [13, 14]. Therefore, Nagayama et al. published promising parameters such as Relative Enhancement (RE), Normalized Iodine Density (NID), and Iodine/VNC Ratio (IVR), which show great potential for differentiating between adrenal adenomas and metastases. However, their group used parameters derived from DECT scans [15]. These parameters has been validated for DECT by Loonis et al. [16].

The introduction of Photon Counting CT (PCCT) has improved clinical imaging capabilities. Unlike conventional detectors that rely on scintillators, PCCT enables the direct measurement of photons, improving spatial resolution and the ability to obtain energy information from photon energy measurements [17]. Bette et al. already demonstrated the potential of PCCT-derived VNC reconstructions for discriminating between adrenal adenomas and metastases [18]. However, their work focused on spectral- and VNC-reconstructions without investigating the potential of parameters as RE; NID or IVR.

The aim of the recent study was to evaluate the potential of PCCT-derived iodine densities and Virtual-Non-Contrast reconstructions, as the potential of parameters as RE, NID and IVR for the differentiation between adrenal adenomas and adrenal metastasis.

## Material/ methods

### Patient cohort

This retrospective single-center study was conducted in accordance with the Helsinki Declaration [19] and was approved by the local institutional review board. The inclusion criteria were as follows: (I) age ≥ 18 years; (II) presence of an adrenal mass (metastases or adenoma), with the diagnosis confirmed either by long-term monitoring of the mass in the context of the underlying disease, correlation via MRI, or biopsy. Adrenal lesions with density values < 0 HU were not included in the study; (III) multiphasic CT of the upper abdomen (including early arterial and portal-venous phases); and (IV) PCCT performed. From December 2021 to September 2023, a total of 53 patients (24 with adrenal metastases and 29 with adrenal adenomas) were retrospectively enrolled in the study.

### CT scan and contrast protocol

A clinical, CE-marked photon counting CT scanner (NAEOTOM ALPHA, Siemens Healthineers AG) was used for all examinations. All scans were performed at 120 kVP tube voltage with adaptive tube current (CARE keV IQ). Rotation time was 0.25 s and pitch was set to 1.5. After pre-monitoring, 60 ml iodine-based contrast agent (Iomeprol 350 mg/ml, Bracco Imaging Deutschland GmbH) was injected at 4.0 ml/second rate followed by 30 ml saline chaser. Image acquisition was started using bolus tracking in the descending aorta at a threshold of 100 HU. After acquisition of early-arterial contrast phase images, additional series were acquired in the portal venous contrast phase (60 s delay).

**Fig. 1:**
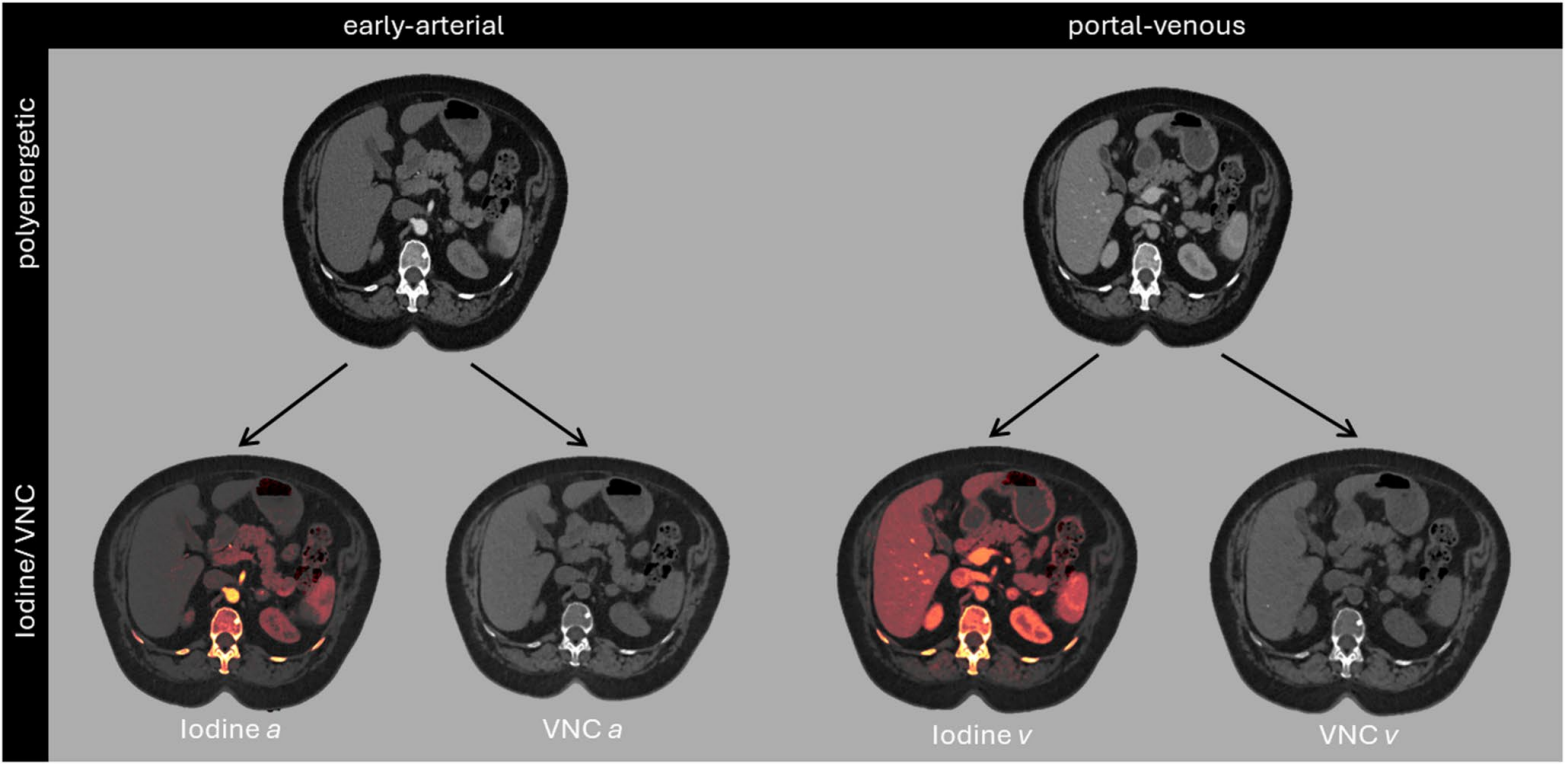
Observed contrast phases and reconstructions

Reconstruction was performed using a dedicated algorithm and a fixed kernel with medium denoising strength (Quantum Plus^®^, QR40 and Q3). Respectively, all images were reconstructed on a 512 × 512 matrix with a slice thickness and section increment of 1.0 mm and 0.5 mm. In the vendor console (syngo.Via, version VB60A) iodine maps and VNC reconstructions of early-arterial (VNCa), and portal-venous (VNCv) contrast phase were generated (Fig. 1).

### Structured measurements

A standardized and structured measurement workflow was performed on polyenergetic (entire CT-spectrum) early-arterial and portal-venous images as on corresponding VNC reconstructions and iodine maps. 2D regions of interest (ROIs) with 1 cm diameter were placed in the adrenal lesions. In case the target structure was smaller than 1 cm, the size of ROIs was adjusted by the reader accordingly. ROIs were placed by a medical student and supervised by a radiologist with 5 years of experience in Abdominal CT and 3 years of experience in PCCT.

### Evaluated parameters

The density values measured in polyenergetic images, VNC reconstructions and iodine maps of the lesions were compared to each other as the iodine concentration determined in the iodine maps. Additionally, the measured values were used to calculate established ratios, which already have shown their potential in DECT [15, 16].

$$\:RE=\frac{{Lesion\:Mean}_{poly}\:-\:{Lesion\:Mean}_{VNC}}{{Lesion\:mean}_{VNC}}*100$$

Relative enhancement (RE) was calculated as mentioned above.

$$\:NID=\frac{{Lesion\:Mean}_{Iodine}}{{Abdominal\:Aorta}_{Mean}}$$

Normalized iodine density (NID) is built by the quotient of the lesions mean density and the mean value of the abdominal aorta.

$$\:IVR=\frac{\text{N}\text{I}\text{D}}{{Lesion\:Mean}_{VNC}}*100$$

Additionally, the Iodine/ VNC ratio (IVR) is the quotient of NID and the lesions VNC mean.

### Statistical analysis

Statistical analysis was performed with R statistics (version 4.1.0) [20]. Mean and standard deviation (SD) were calculated for quantitative variables, percentages (%) were calculated for categorical variables. Comparison tables were created with the tableone package (version 0.13.2). Regarding the patient cohort, p-values for age and the lesions diameter were calculated using t-test. F-test was performed before to compare the variances. P-values for number of females and localisation of the lesion were calculated using chi-square-test. Boxplots were created with ggplot2 (version 3.3.5). The groups of adrenal adenoma and metastases were compared suing Mann-Whitney-U-Test. P-values < 0.05 were regarded as significant. Receiver operating characteristic (ROC) curves were calculated using the pROC package. Thresholds for the curves were calculated using Youden-Index.

## Results

### Patient cohort

In accordance with the mentioned inclusion criteria the presented retrospective single-center study included 53 patients (24 female) with adrenal lesions. In total 29 adrenal adenomas and 24 adrenal metastases were included. No significant differences between gender or laterality of the lesion were reported. Mean diameters of adenomas and metastases however were significantly different. Detailed information about the patient cohort is given in Table 1.

**Table 1:** Patient collective overview. (1) p-values for age and the lesions diameter were calculated using t-test. F-test was performed before to compare the variances. (2) p-values for number of females and localisation of the lesion were calculated using chi-square-test. p-values < 0.05 were taken as significant

Group	Total	Adenoma	Metastases	*p*-value
n	53	29	24	-
Age Mean				
Mean	66.9	67	66.7	0.93^1^
SD	11.4	13	8.9	-
Number of Female				
Total (n)	24	14	10	0.63^2^
Percentage (%)	45.3	48.3	41.7	-
Lesion Right				
Total (n)	20	6	14	0.08^2^
Percentage (%)	37.7	20.7	58.3	-
Diameter of Lesion [mm]				
Mean	19	15.4	23.4	< 0.05^1^
SD	8.5	4.9	9.8	-

### Density mean values of adrenal lesions

**Fig. 2:**
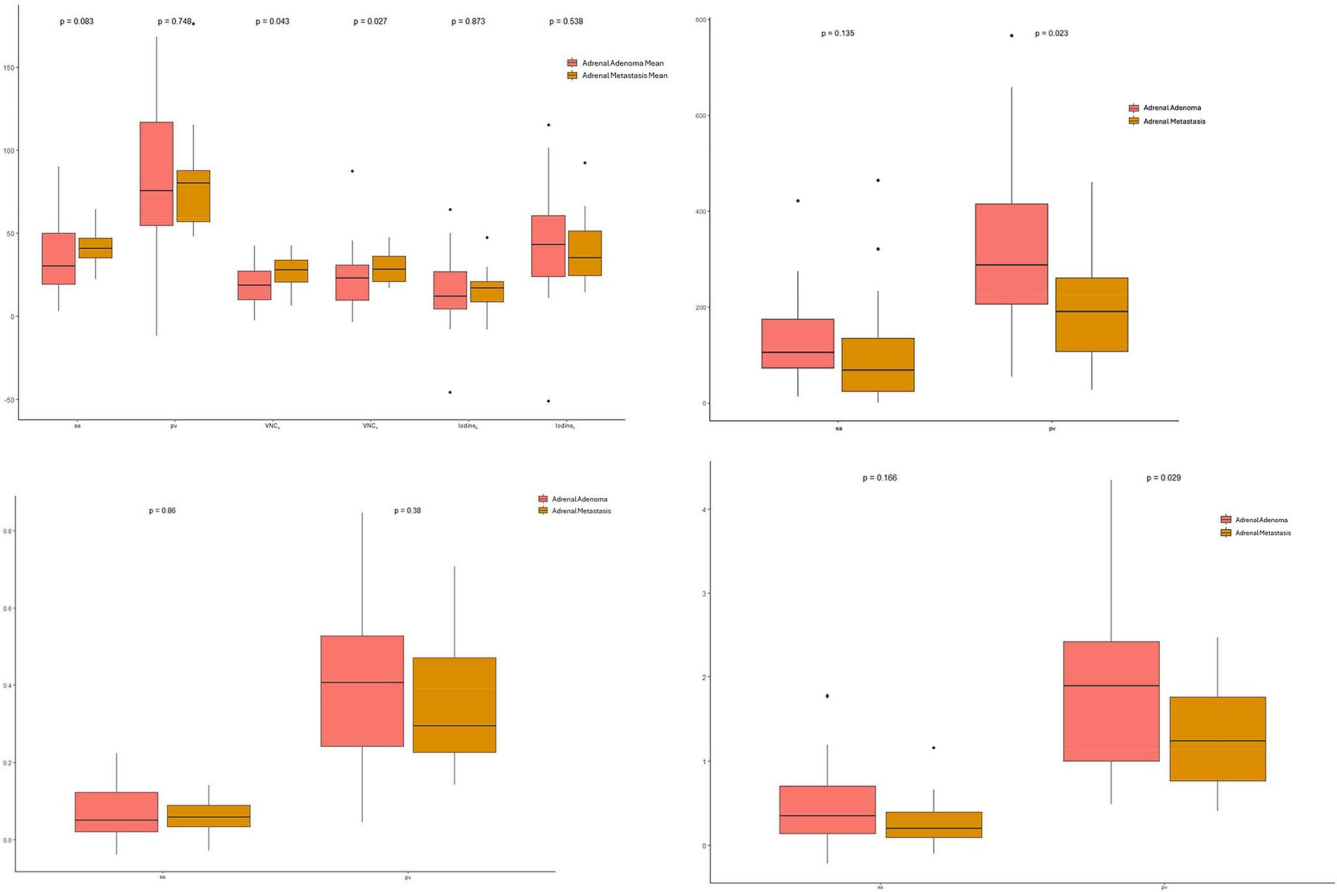
**A**: Mean values measured in adrenal lesions. Adrenal adenoma is represented by the red plot, adrenal metastases by the yellow blot. P-values were calculated using Mann-Whitney-U-test. p-values < 0.05 were regarded as significant. Abbreviations: early-arterial (ea), portal-venous (pv), Virtual non contrast derived from ea (VNC_a_), Virtual non contrast derived from pv (VNC_v_), Iodine maps derived from ea (Iodine_a_), Iodine maps derived from pv (Iodine_v_). **B**: Relative enhancement. Adrenal adenoma is represented by the red plot, adrenal metastases by the yellow blot. P-values were calculated using Mann-Whitney-U-test. p-values < 0.05 were regarded as significant. Abbreviations: early-arterial (ea), portal-venous (pv). **C**: Normalized iodine density: Adrenal adenoma is represented by the red plot, adrenal metastases by the yellow blot. P-values were calculated using Mann-Whitney-U-test. p-values < 0.05 were reported as significant. Abbreviations: early-arterial (ea), portal-venous (pv). **D**: Iodine/ VNC-ratio: Adrenal adenoma is represented by the red plot, adrenal metastases by the yellow blot. P-values were calculated using Mann-Whitney-U-test. p-values < 0.05 were given as significant. Abbreviations: early-arterial (ea), portal-venous (pv)

Looking at the mean values of the adrenal lesions there can be observed significant differences between adenomas and metastases in the VNC reconstructions. They differ significantly in ea-derived VNC (*p* = 0.043) and in pv-derived VNC (*p* = 0.027). No significant differences were observed looking at the conventional image data and at the iodine maps (Fig. 2A; Table 2).

**Table 2:** Mean values of adenomas and metastases in early-arterial (ea) and portal-venous (pv) contrast phase as in ea and pv derived Virtual-non-Contrast (VNC) reconstructions and iodine maps

	ea	pv	VNC_a_	VNC_v_	iodine_a_	iodine_v_
Adrenal_Adenoma_Mean (mean (SD))	34.88 (22.90)	83.21 (41.22)	20.93 (14.46)	21.64 (17.81)	15.55 (21.59)	43.52 (32.47)
Adrenal_MTX_Mean (mean (SD))	41.58 (10.13)	79.30 (28.39)	26.53 (10.29)	28.26 (8.82)	27.70 (61.09)	38.87 (18.76)

### Relative enhancement

The Relative Enhancement ratio (RE) of the lesions showed a significant difference in the portal-venous contrast phase (*p* = 0.023). RE calculated from early-arterial scans showed no significant differences between adenomas and metastases (Fig. 2B).

### Normalized iodine density

Results of the Normalized Iodine Density (NID) are shown as boxplots in Fig. 2C. Both the NID calculated from the ea scans (*p* = 0.86) and that from the pv scans (*p* = 0.38) showed no significant differences between adenomas and metastases.

### Iodine/ VNC ratio

Iodine/ VNC Ratio (IVR) calculated from density values measured in pv scans showed significant differences between adenomas and metastases (*p* = 0.029). IVR calculated from density values measured in ea scans (*p* = 0.166) showed no significant differences between the entities (Fig. 2D).

### Comparison of the different methods of discrimination

**Fig. 3:**
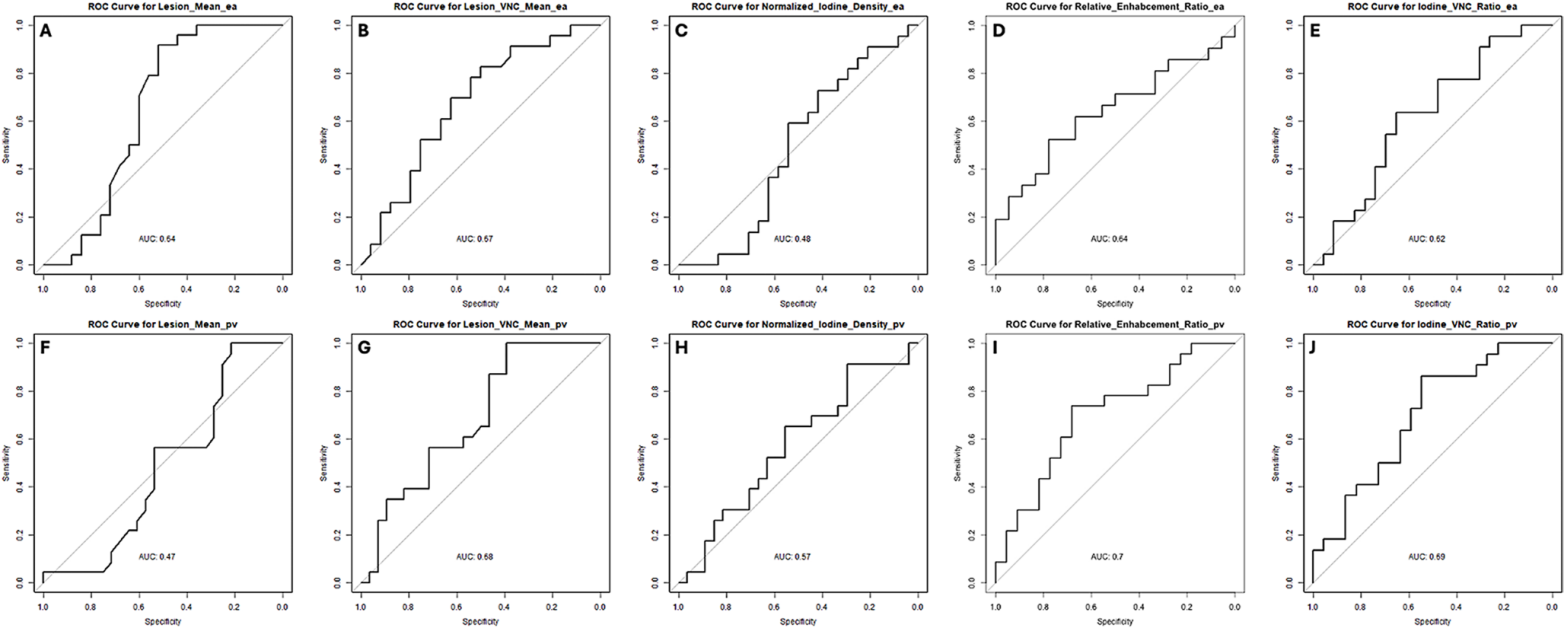
Comparison between the AUCs of the applied parameters. X-axis shows specificity and y-axis shows sensitivity. Thresholds for the curves were calculated using Youden-Index. **A-E** show curves for early-arterial contrast phases (ea). **F-J** show curves for portal-venous contrast phases (pv). Results for conventional scans (**A + F**), virtual non contrast (VNC; **B + G**), Normalized Iodine Density (**C + H**), Relative Enhancement Ratio (**D + I**) and Iodine-VNC-Ratio (**E + J**) are shown

To compare the different measurements and ratios additional ROC analyses has been carried out. The corresponding results are illustrated in Fig. 3; Table 3. The highest AUC (0.7) with a sensitivity of 0.52 and a specificity of 0.78 was detected for RE calculated of density values measured in pv scans. The lowest AUC was observed for the density measurements in pv contrast phase (0.47). For all calculated ratios (RE, NID and IVR) the AUCs calculated from pv measurements were higher than the ones calculated from ea measurements.

**Table 3:** Parameters of the receiver operating characteristic (ROC) curves for differentiating adrenal adenomas and metastases. Thresholds for the curves were calculated using Youden-Index

Method	Threshold	AUC	Sensitivity	Specificity
Lesion_Mean_ea	31	0.64	0.92	0.52
Lesion_Mean_pv	47.5	0.47	1	0.21
Lesion_VNC_Mean_ea	17.9	0.67	0.83	0.50
Lesion_VNC_Mean_pv	15.2	0.68	1	0.39
Relative_Enhabcement_Ratio_ea	70.4	0.64	0.52	0.78
Relative_Enhabcement_Ratio_pv	247.6	0.7	0.74	0.68
Normalized_Iodine_Density_ea	0.04	0.48	0.73	0.42
Normalized_Iodine_Density_pv	0.50	0.57	0.91	0.30
Iodine_VNC_Ratio_ea	0.29	0.62	0.64	0.65
Iodine_VNC_Ratio_pv	1.88	0.69	0.86	0.55

## Discussion

The recent study underlines the great potential of PCCT in oncological imaging by enabling differentiation between adrenal adenomas and adrenal metastases, utilizing PCCT-derived image parameters from routine workflows, especially RE, which can be used in contrast enhanced scans to differentiate between adrenal adenomas and adrenal metastases.

While adrenal adenomas are typically smaller compared to metastases [21, 22], a significant difference between the adenomas (mean diameter: 15.4 mm) and metastases (mean diameter: 23.4 mm) included in the recent study was observed. Density values obtained from ea- and pv-contrast enhanced scans showed no significant differences between adenomas and metastases, which underlines the challenge of differentiating AINs in conventional contrast enhanced CT-imaging. However, density values measured from VNCa (*p* = 0.043) and VNCv (*p* = 0.027) exhibited significant differences between adenomas and metastases, possibly owing to their low microscopic or macroscopic fat [23]. Compared to further DECT-based studies like Loonis et al. [16] the recent study showed lower diagnostic performance of VNC reconstructions. For instance, this may be due to different scan parameters and calibrations. Furthermore, there is need for multicentric studies with lager cohorts. Several studies were able to demonstrate that PCCT-derived VNC reconstructions may substitute true non-contrast images in many cases [24–26]. However, the overall mean values of adenomas were in VNCa (20.93 HU) and VNCv (21.64 HU) higher than the suggested cut-off (20 HU) for benign adrenal lesions in unenhanced imaging suggested by the EURINE-ACT study [21]. Bette et al. demonstrated that VNC algorithms overestimate CT values compared to true-non-contrast images. Accordingly, they suggest an adapted threshold of 26 HU to differentiate adrenal adenomas from metastases [18]. In line with these findings the recent study showed density values < 26 HU for adrenal adenomas in VNCa and VNCv and values > 26 HU for adrenal metastases in VNCa (26.53) and in VNCv (28.26), results given in Table 2. Due to the limited differences in VNCa and the large standard deviations in the VNC measurements, there remains potential for improvement and a need for additional factors to achieve better differentiation of adrenal lesions. Concerning the iodine maps, no significant differences were observed between adrenal adenomas and metastases.

Several studies by Nagayama et al. and Loonis et al. introduced and validated factors as relative enhancement (RE), normalized iodine density (NID) and iodine/ VNC ratio (IVR) in DECT [15, 16]. However, there are no studies examining these promising parameters for the PCCT. These factors are calculated as described in the methods section. In this context, alongside the polyenergetic images, which are standard in diagnostics, RE also takes VNC reconstructions into account by computing a quotient based on the difference between the polyenergetic and VNC measurements relative to the VNC measurement. RE calculated from pv scans show significant differences between adenomas and metastases and show the highest AUC in our study. However, RE calculated from ea images show no significant differences between adenomas and metastases, this might be due to the fact that the lesions show no relevant contrast media uptake in the ea contrast phase, and this effects the quotient. Nevertheless, the results of the RE calculated from pv scan show a higher AUC than the conventional measurements in VNC reconstructions. Studies on DECT derived data sets from Nagayama et al. and Loonis et al. showed the same improvement by calculating RE [15, 16]. In NID no significant differences were observed between adenomas and metastases. However, NID can be used to calculate IVR, representing the quotient of NID and density in VNC. Looking at the results there is no significant difference between the lesions in ea contrast phase, which may due the small iodine uptake in this contrast phase. Measurements of pv contrast phase show significant differences between adenomas and metastases.

In total the recent study showed that PCCT derived VNC reconstructions, as RE and IVR from pv contrast phase can be used to differentiate between adrenal adenomas and metastases. These results are in line with the preliminary data from DECT [15, 16]. Given the recent results the best diagnostic performance was observed in RE (Sensitivity: 74%, Specificity: 68%). Best sensitivity was shown in VNCv density (100%) compared with a low specificity (39%). IVR derived from pv contrast phases showed also a good sensitivity (86%) with reduced specificity (55%) compared to RE. Since only two measurements and the VNC reconstructions are required for RE, it could be calculated quickly in clinical routine and provide an initial orientation for AINs. However, dedicated CT protocols for adrenal imaging can reach sensitivities and specificities up to 98% and 92% [27]. Additionally, chemical shift MRI allows for the calculation of the signal intensity index (SII), which has been shown to discriminate adenomas from metastases with an accuracy of 100% [28]. In this context, the spectral imaging measurements and calculations derived from DECT and PCCT are not yet capable of replacing these established techniques. Nevertheless, they offer a straightforward and efficient initial assessment without requiring additional imaging. Compared to MRI, PCCT provides superior spatial resolution and contrast-to-noise ratio, which enhances the visualization of small or low-contrast lesions [29, 30]. MRI, while effective in characterizing adrenal masses, particularly lipid-rich adenomas, can be limited by its lower spatial resolution and longer acquisition times [31, 32]. PCCT’s ability to generate virtual non-contrast (VNC) images and material decomposition techniques further aids in the accurate characterization of adrenal lesions, reducing the need for additional imaging studies [18, 33].Accordingly, the Canadian Urological Association and American Urological Association guidelines recommend non-contrast CT as the first-line imaging for incidental adrenal masses, with MRI as a secondary option for indeterminate cases [31]. PCCT’s advanced capabilities align well with these guidelines, offering improved diagnostic accuracy and potentially reducing the need for follow-up MRI.

In routine practice, PCCT can streamline workflows by providing comprehensive imaging data in a single scan, reducing the need for multiple imaging modalities. Its high spatial resolution and spectral capabilities allow for detailed tissue characterization, which is crucial for accurate diagnosis and treatment planning [34, 35]. Additionally, PCCT’s potential for reduced radiation dose is particularly beneficial for patients requiring frequent imaging [29, 30].

For clinical application, however, the small number of preliminary studies with comparatively small numbers of cases and the inferior specificity and sensitivity of special imaging represent a strongly limiting factor, so that dedicated imaging of the adrenal glands cannot simply be dispensed with as part of good clinical practice.

One of the main limitations of the present study is the relatively small patient cohort, which is primarily due to the limited number of PCCT examinations conducted to date. Furthermore, as the scans were obtained from routine clinical practice, true non-contrast (TNC) images were not available. This represents an additional limitation, as TNC imaging could have been used to validate the VNC reconstructions.

Despite these constraints, the current study serves well as a proof-of-concept and provides a valuable foundation for future research. In particular, expanding the sample size through multicentre studies would be an important next step. It would also be beneficial to include studies that contain native scans, which could also reach a sufficient cohort size through collaborative, multicentre efforts.

Overall, the introduction of PCCT represents a significant advance in adrenal imaging, offering the capability for adrenal mass characterization from routine scans which can be expected to improve patient safety and decrease overall radiation exposure.

## Data Availability

No datasets were generated or analysed during the current study.

## Abbreviations

AIN: Adrenal incidentalomas
AUC: Area under the curve
DA: Descending aorta
DECT: Dual Source CT
ea: Early-arterial
IVC: Inferior vena cava
IVR: Iodine to VNC ratio
L1: First lumbar vertebral body
NID: Normalized iodine density
PCCT: Photon counting CT
PM: Psoas muscle
pv: Portal-venous
PV: Portal vein
RE: Relative enhancement
ROC: Receiver operating characteristic
ROI: Regions of interest
SAT: Subcutaneous adipose tissue
SII: Signal intensity index
VNC: Virtual-Non-Contrast
VNCa: VNC reconstructions of early-arterial contrast phase
VNCv: VNC reconstructions of portal-venous contrast phase
